# Development and Function of the Intestinal Microbiome and Potential Implications for Pig Production

**DOI:** 10.3390/ani9030076

**Published:** 2019-02-28

**Authors:** Tanya L. Nowland, Kate J. Plush, Mary Barton, Roy N. Kirkwood

**Affiliations:** 1 School of Animal and Veterinary Sciences, The University of Adelaide, Roseworthy SA 5400, Australia; roy.kirkwood@adelaide.edu.au; 2 SunPork Solutions, Shea-oak Log SA 5371, Australia; kate.plush@sunporkfarms.com.au; 3 School of Pharmacy and Medical Sciences, University of South Australia, Adelaide SA 5000, Australia; mary.barton@unisa.edu.au

**Keywords:** intestinal microbiota, neonatal environment, management, piglet, performance

## Abstract

**Simple Summary:**

Piglet preweaning mortality is a major economic loss and welfare concern for the global pork industry, with the industry average sitting at approximately 15%. As such, novel methods for reducing this mortality are needed. Since research into the intestinal microbiota has provided advances in human health, in particular the impact of early life factors, it was the logical next step to synthesise the existing literature to determine the potential relevance to the pig industry. It is evident from the literature that this area of research provides promising results. However, a large gap within the literature currently exists within the lactation period in pigs. Since optimal development within early life is proving to be critical for human infants, it is crucial that further research is invested into understanding the impact of early life events on a piglet’s microbiome. It is hoped that this review will enable access to critical information for those interested in the microbiome and its potential for improving herd health on the farm.

**Abstract:**

The intestinal microbiota has received a lot of attention in recent times due to its essential role in the immune system development and function. Recent work in humans has demonstrated that the first year of life is the most critical time period for microbiome development with perturbations during this time being proven to have long term health consequences. In this review, we describe the literature surrounding early life events in humans and mice that contribute to intestinal microbiota development and function, and compare this to piglets predominantly during their lactation period, which focuses on the impact lactation management practices may have on the intestinal microbiota. Although extensive research has been conducted in this area in humans and mice, little research exists in pigs during perceivably the most critical time period of development, which is the lactation period. The research reviewed outlines the importance of appropriate intestinal microbiota development. However, further research is needed in order to understand the full extent routine farm practices have on a piglet’s intestinal microbiota.

## 1. Introduction

At parturition, the neonate is exposed to a range of microorganisms and this initial exposure forms the basis of the microbiome. A microbiome is a community of microorganisms that together have a mutualistic relationship with the host [1]. There are multiple niches on the host that have their own characteristic microbiome, e.g., the skin, mouth, urogenital tract, and all levels of the gastrointestinal tract (GIT) [2,3,4], with each characteristic microbiome maintaining a diverse and relatively stable population of bacteria, archaea, fungi, and viruses [2,3,4]. The types of microorganisms that make up the gastrointestinal microbiota play an integral role in host metabolism, the development of a healthy GIT, and immune and neural system development [5,6]. As such, the health of offspring largely depends on the microorganisms that the body is exposed to throughout life. Studies in humans have demonstrated that the first year of life is the most critical time period for microbial acquisition and development, with disruptions to the microbiome during this time causing long-term consequences [7]. Some of the consequences observed in humans are gastrointestinal and systemic disorders including inflammatory bowel disease, necrotizing enterocolitis, eczema, obesity, autoimmunity, asthma, and autism [8,9,10]. These findings serve to illustrate the power of an appropriate intestinal microbiota and have initiated the idea that fostering a healthy microbiome through optimal initial colonisation may improve health and limit disease.

Finding novel methods for improving health on the farm is of particular interest to the pork industry since pre-weaning mortality is a major issue with the industry average being around 15% [11,12,13]. To date, a large quantity of research exists surrounding the development of methods for reducing these mortalities. However, it has proven to be a complex issue with little progress [11,13,14]. Research investigating the impact of routine farm practices on piglet microbiome is limited, with the majority of research in this area focusing on the microbiome in pigs as a model for humans and in piglets post weaning [15,16,17]. Given our understanding of the importance of early microbial colonisation and GIT health in humans, it is reasonable to suggest that fostering a healthy microbiome may contribute to piglet viability and survival. This review summarises studies examining initial microbial acquisition and development, the impact of different factors on the microbiome, and the impact of the microbiome on health and methods of microbiome manipulation.

## 2. Acquisition of the Microbiome 

### 2.1. Pre-Partum Microbial Acquisition

The development of the microbiome has long been thought to originate at birth when the fetus transits from a supposedly sterile environment within the amniotic sac through the birth canal, into a microbially dense environment. However, recent studies in mice and humans question these claims and have demonstrated the presence of bacteria within the amniotic fluid of pregnant mice and the meconium of infants, which suggests some colonisation in utero [18,19]. Whether in utero colonisation occurs in food species such as pigs remains unknown. However, the differences in placentations (haemochorial vs. epitheliochorial) would likely impact the ability for microbial transfer. Additional microbial colonisation occurs during the parturition process when microorganisms colonise the mucus membranes and skin epithelia. Factors such as transit time and mode of delivery have an influence on the colonising microbial populations [20]. Neonates born vaginally are colonised by microorganisms that are similar to their mother’s vaginal microbiota, while those delivered by caesarean section (C-section) are colonised by bacterial communities similar to the mother’s skin microbiota [21,22,23]. The impact of the delivery method on the infant has been extensively studied over recent years with accumulating evidence suggesting that C-sections that delivered human infants have reduced microbial richness and diversity compared to those delivered vaginally [23,24]. It is this reduced diversity that is suggested to be the main cause of the increased incidence of allergic disease often seen in human infants delivered by C-section [24,25]. While delivering pigs via C-section is not a likely option within the pork industry generally, this research provides evidence that the microorganisms harbored at birth have long-term implications for health. This is of particular interest since C-sections are often used when establishing new farms, especially at the nucleus level where high health is the required outcome. The implications of an ‘abnormal’ microbiome may be detrimental to this requirement.

### 2.2. Post-Partum Microbial Acquisition

The post-partum GIT microbiota has three essential roles, which include protective, metabolic, and trophic roles [26]. First, the microorganisms act as a barrier against pathogenic organisms by competitive exclusion. Then, they aid in digestion and metabolism of colostrum and milk, they break down toxins and drugs, synthesise vitamins, and absorb ions. Lastly, they support the growth and differentiation of the epithelial cells lining the intestinal lumen and support homeostasis of the immune system [26]. Human post-natal factors such as feeding type (breast vs. formula), maternal weight gain, stress and prenatal and perinatal antibiotic use all influence the populations of bacteria colonising, which alters the way the microbiota performs these functions [7,27,28]. Although the microbiome is influenced via a variety of external factors in pigs, two predominant immediate postnatal factors that determine initial postnatal microbial colonisation are likely colostrum, milk quality, and the neonatal environment.

#### 2.2.1. Colostrum

The importance of colostrum and milk for human and animal health has been extensively studied with a number of reviews available [9,27,28,29,30]. Maternal milk provides energy, nutrients, and bioactive compounds such as immunoglobulins, cytokines, chemokines, growth factors, hormones, and antibodies that directly influence development [27,29]. It also contains other compounds such as peptides, lactoferrin and other whey proteins, oligosaccharides, and a large number of bacteria [27]. Maternal milk is an important postnatal element for establishing an appropriate intestinal microbiota [31]. Studies in humans have demonstrated that being breast-fed is associated with a lower incidence of diabetes, obesity, celiac disease, multiple sclerosis, and asthma [29,32]. These associations are primarily driven by the protective effects of milk against early infections, its anti-inflammatory properties, antigen specific tolerance induction, and regulation of the infant’s microbiome [29]. While clearly important in humans, the significance of the enteromammary axis in food animals is likely limited by the relatively short lacations, but likely becomes more significant with longer lactations. In pigs, longer lactations and higher weaning weights have been associated with improved health outcomes and fewer days to market. A role for the enteromammary axis in these benefits from longer lactations cannot be discounted.

The microbiome of the breastfed infant is very different from that of formula fed infants [21,33]. There is also a demonstrated specificity between the microbiome and suckling with a study in rodents demonstrating that milk cells contain a number of bacterial DNA signatures found in maternal peripheral blood mononuclear cells during pregnancy and lactation, which suggests bacterial translocation [34]. Other studies in humans and mice have suggested that this is a result of dendritic cells sampling the luminal microbiome and translocating it into the milk [29,34,35]. Although currently speculative, it seems reasonable to suggest a similar differential effect of maternal milk and formula would also occur in pigs and should be considered when providing supplemental nutrition to compromising pigs, such as those with low birth weights.

Compared to humans, pigs are born with relatively low body energy stores and are immunologically naive due to the epitheliochorial nature of the porcine placenta [36]. This means that, for the piglet, the consumption of colostrum immediately after parturition is essential to survival [37]. Colostrum not only provides a supply of warmth, energy, and immunity, it also enables the establishment of commensal microbes. The ability to acquire colostrum is largely dependent on piglet weight at birth [38] and, as the industry pushes towards improved sow prolificacy and a greater number of newborn piglets per litter, the proportion of low birth weight piglets is increased [38]. Morissette et al. [31] suggested colostrum and milk intakes (as measured by weight gain) within the first two weeks of life influenced the development of the microbiota. High weight gain piglets have higher levels of *Bacteroidetes*, *Bacteroides*, and *Ruminoccocaceae* and lower proportions of *Actinobacillus porcinus* and *Lactobacillus amylovorus* compared with low weight gain piglets. These data suggest that the quantity of milk ingested within the first two weeks of life has the potential to not only impact weight gain but also influence long-term animal health and performance via the microbial populations colonising. Low birth weight piglets do not reach the udder as fast and have reduced competitiveness for teats [12,38], and a potential lack of maturity of the GIT may also impact the outcomes observed. It is evident that further research is required in order to establish the etiologic influence colostrum acquisition has on the development of the microbiome in piglets. Although an interaction exists between milk consumption and the microbiome, the impact of the quantity and quality of the milk obtained and its effects on the microbiome is yet to be investigated in the piglet.

#### 2.2.2. Environment

Both pathogenic and non-pathogenic bacteria are ubiquitous in the environment. It is the combination of the environment, diet, and genetics that determine which microbes colonise the epithelial surfaces of the body [7,27,30]. In humans, it is relatively difficult to completely eliminate the confounding factors of differences in diet, genetics, gestation, and the delivery method from the impact of the environment alone. As such, animal studies have been conducted in order to fill these gaps. 

In mice, it has been demonstrated that immunological development is largely dependent on the initial GIT microbial colonisation, which is determined by the environment. Cahenzli et al. [8] demonstrated that mice that were germ-free at birth and that were maintained in a germ-free environment had an increased antigen-induced oral anaphylaxis incidence, which demonstrates the importance of an appropriate intestinal microbial stimulus for immune system development. In pigs, the influence of low hygiene (farm housed, sow-fed) or high hygiene (isolator housed, milk formula-fed) environments influenced piglet immunological development. Piglets reared on the sow have a more diverse intestinal microbiota than the siblings reared in isolators [39]. It is impossible to determine the direct effect nutrition has on this. However, the latter study further corroborates the findings from previous studies, which indicate that the microorganisms that colonise the GIT influence immune development and subsequent health. 

When considering the development of the microbiome in pigs, an understanding around the piglet postnatal environment is essential. Since pigs are produced within an intensive production system where they are housed in pens in contact with the mother’s feces, skin, and mucosal surfaces until weaning, it is likely that the microbiome of a newborn piglet is largely dependent on the sow. When considering the opportunity for microbial manipulation through early life exposure, this may provide an effective arena, with studies suggesting that the pre-weaning period is critical for appropriate colonisation and immune system development [8,21,30]. Further investigations of the lactation period should prove fruitful. The development and variation of the microbiome in pigs is starting to gain understanding since a number of studies are investigating this [40,41]. However, relatively little is known about what impact general farm practices, including sow nutrition and parity, farrowing crate cleanliness, sow skin and udder cleanliness, piglet fostering, iron and penicillin injections at 24 h old, and age of weaning are having on the microbiome and individual piglet performance. 

## 3. Impact of Different Factors on the Microbiome 

The initial colonising bacteria largely drive microbiome establishment and development. However, the microbiome is a dynamic system that is continuously changing and is influenced by a variety of factors. Some of these factors include antibiotic use, stress, diet, age, and the rearing environment [7,27,28]. Previous studies in humans have suggested that the most important period for microbial establishment is the first 1 to 3 years of life since it is during this time that the microbiome is more dynamic and susceptible to change [42,43]. Disruption or dysbiosis during this period result in disease [8,44,45]. Thereafter, the microbiome changes toward a more adult-like state where it becomes more stable and resistant to change [42,43]. Accumulating evidence suggests that the shift in the microbial state may be attributed to the transition from a primarily liquid milk diet to one that relies on solid food [9,43]. From these data values, it can be assumed that, in the case of the pig, the most critical time for microbial establishment would be prior to weaning while they still maintain a predominantly milk-based diet. As such, the practices undertaken during lactation should be critically reviewed in order to establish the potential impact they are having on the microbiome with the ultimate goal promoting the establishment of a healthy microbiome. 

### 3.1. Antibiotics 

Antibiotic use during the pre-natal and post-natal period has been demonstrated to negatively impact GIT microbial diversity and increase the number of resistant bacteria [46,47,48]. Antibiotics are commonly used for the control of pathogenic bacteria. However, they are non-specific and have the potential to perturb beneficial commensal bacteria in the GIT and elsewhere [49,50]. In humans, when administered during the early postnatal period and while the initial microbial establishment is occurring, these disruptions can lead to overgrowth of pathogenic bacteria and to long-term health problems such as asthma, necrotising enterocolitis, and late-onset sepsis [5,49,51]. 

In recent times, the importance of populating a healthy microbiome has become increasingly evident. As a result, a multitude of reviews surrounding the use of antibiotics on the microbiome have been conducted [26,46,49,50]. What can be taken collectively from these reviews is that microbial disruption during the perinatal period has detrimental effects on microbial establishment and metabolism, which often leads to long-term health problems. When investigating the effect of antibiotics in the pig specifically, a similar effect on microbial diversity and quantity is observed. Gao et al. [15] investigated the time-course effect of antibiotics on microbial composition and metabolism in pigs fed a standard diet with or without antibiotics. Their findings support the human literature where antibiotic administration leads to changes in microbial GIT communities and metabolism. These differences are noticeable as soon as two days in the ilium and seven days in the faeces. Similarly, studies conducted by Looft et al. [52,53] demonstrated that in-feed antibiotics for piglets caused divergence in microbiome membership and reduced microbial population quantity and diversity. These studies also demonstrated that *Escherichia coli* populations in the ileum increased with antibiotic exposure. Antimicrobial resistance genes to antibiotics that were not administered were identified. Furthermore, a study conducted by Kim et al. [53] demonstrated similar changes in microbiota populations in pigs when administered antibiotics were in the feed. It is common practice globally for piglets to receive antibiotic treatment at 24 h of age but little research has investigated its consequences with regards to the microbiome.

### 3.2. Stress

There is a growing body of evidence linking the GIT microbiota to the central nervous system function [26,54,55]. O’Mahony et al. [56] observed that rat pups exposed to maternal separation stress for three hours daily from two to 12 days of age and then exposed to a novel stressor had an increased number of faecal boli, increased plasma corticosterone levels, an increased visceral sensation, and an altered faecal microbiota when compared to undisturbed rats. Additionally, Bailey et al. [57] found that mice exposed to a social disruption stressor had large shifts in the microbiota community structure, which decreased the relative abundance of bacteria genus *Bacteriodes* while increasing the relative abundance of *Clostridium*. Additionally, the stressor also increased circulating levels of IL-6 and MCP-1, which correlates with changes in three bacteria genera *Coprococcus*, *Pseudobutyrivibrio*, and *Dorea*. Additional studies have also demonstrated that chronic stress affects the abundance and diversity of the GIT microbiota, which have long-term effects on the immune system [26,49,54]. 

Within the pork industry, weaning would be the most commonly studied event that causes stress in pigs. Weaning is a multifactorial stressor, including environmental, social, nutritional, and psychological disruptions [58]. The stress associated with weaning is a welfare concern and it causes a reduction in growth during the first days following weaning, which results in economic loss with increased days to market [58,59]. This reduced weight gain post weaning, commonly referred to as the ‘post weaning growth check,’ is thought to be a result of the reduced intestinal integrity caused by stress, which causes leaky gut and diarrhoea as well as an increased susceptibility to colonisation with pathogenic bacteria such as *E. coli* [58,59,60]. Experiments in animals suggest that increased cortisol levels that accompany stress are the main driver for this, which increases gut permeability and bacterial lipopolysaccharide leakage across the intestinal wall [61,62]. The social stress from weaning is exacerbated by litters being mixed.

While stress affects the microbiota, evidence suggests that there is a bi-directional communication between the gut and the brain, which means that the intestinal microbiome can influence the animal’s susceptibility to stress or anxiety [54,55,63]. When comparing germ-free (GF) mice with specific-pathogen free (SPF) mice, Sudo et al. [64] demonstrated that GF mice had elevated plasma adrenocorticotropic hormone and corticosterone levels in response to a restraint stress. GF mice also exhibited reduced brain-derived neurotrophic factor expression levels in the cerebral cortex and hippocampus. Furthermore, when the GF mice were reconstituted with *Bifidobacterium infantis*, the exaggerated HPA stress response observed could be reversed. Other such cases of this ‘reversal’ in behavior through microbial supplementation have been demonstrated. Messaoudi et al. [65] found that anxiety-like behavior was reduced in rats and physiological distress (depression and anger-hostility) was reduced in humans when given a combination of *Lactobacillus helveticus* R0052 and *Bifidobacterium longum* R0175. The ability to alter an animal’s response to stress by introducing different GIT bacteria is new and, although the results from these trials are promising, more research in this area is needed. Influencing the intestinal microbial composition in order to improve productivity and health are the ultimate objectives for the future and an improved ability to cope with stress would be beneficial to productivity.

Few studies to date have demonstrated the impact of prenatal stress on microbial establishment. Zijlmans et al. [66] demonstrated a link between maternal stress and microbiota colonization. Mothers who were identified as having high cumulative stress (high reported stress and high cortisol concentrations) during pregnancy had a significantly higher relative abundance of proteobacterial groups that are known to contain pathogens (i.e., *Escherichia*, *Serratia*, and *Enterobacter*), while beneficial bacteria such as *Lactobacillus*, *Lactococcus*, *Aerococcus*, and *Bifidobacteria* were reduced. Additionally, Gur et al. [67] not only demonstrated that prenatal stress resulted in different placental microbes in mice offspring, but also that prenatal stress led to long-term differences in behaviour and cognition with increased anxiety like behaviour in female mice and decreased social interaction in male mice. Within the pork industry, more research has been conducted into gestational group housing and farrowing accommodation in order to identify optimal housing for reduced sow stress and improved piglet welfare and survival. However, to our knowledge, no research to date has investigated the effect of housing during both the gestational and the pre-farrow period on the sow or piglet’s microbiome and subsequent health. These studies provide precedent for further investigation into this area, especially in the case of intensive production systems with the increased risk for high stress.

### 3.3. Age and Diet

Diet represents one of the major factors contributing to intestinal microbial colonisation [68]. This is evident from research showing marked differences in the GIT microbiota community structure in pigs after only two weeks of feeding different experimental diets [69]. Similarly, a gradual taxonomic and functional rearrangement of the bacterial community in feces after feeding four different diets varying in protein source, calcium, and phosphorus concentration has been recorded [70], which indicates the importance of diet on microbial population modulation.

The largest and most dynamic change in microbiome transition, however, is during the weaning period. As such, studies have investigated the influence of weaning from an exclusive milk diet to a solid food diet on the microbiome [28,71]. In pigs, multiple authors have demonstrated that the microbiota of suckling piglets predominantly contained *Bacteroides*, *Oscillibacter*, *Escherichia/Shigella*, *Lactobacillus*, and unclassified *Ruminococcaceae* genera [40,72]. However, after weaning, the bacterial diversity increased linearly to be predominantly *Acetivibrio*, *Dialister*, *Oribacterium*, *Succinivibrio*, and *Prevotella* genera. In contrast, others have observed a reduction in GIT microbial diversity until 11 days post weaning. Microbial diversity followed the trends observed by the previously mentioned studies [16]. These data provide an explanation for the reduction in weight gain and presence of diarrhea often observed in piglets post weaning. The differences observed between studies may be attributed to the fact that weaning ages and sample time points varied between studies. Hu et al. [16] conducted the only study to collect samples within eight days of weaning. This might indicate that the previous studies may have experienced the same drop in diversity, but they had no means for observing it since they had not investigated the microbiota at an early enough time point. Studies like those of Hu et al. [16] provide insights into possible methods that could be implemented to improve microbial diversity around weaning to enable increased stress tolerance for piglets. 

Studies investigating the role diet has on modulating microbial populations and health provide promise for possible investigations in pigs. For example, an increase in fiber in the diet changed the GIT microbiota and increased protection against dysbiosis in mice, which prevented the development of hypertension and heart failure in hypertensive mice [73]. Studies in pigs are beginning to follow this trend and investigate the effect diet has on the microbiota and health. Heo et al. [74] fed piglets different protein levels post weaning and challenged them with an enterotoxigenic strain of *Escherichia coli*. They determined that those animals that were fed a reduced protein diet, had a reduced incidence of post weaning diarrhea in the face of an *E. coli* challenge. Unlike the previous authors, Qiu et al. [17] examined the microbiome and found that 65-day-old gilts fed diets with reduced crude protein levels had a shift in microbial composition in the ileum, which lead to enhanced microbial fermentation and short chain fatty acid production. Overall, these data support the suggestion that intestinal microbial colonisation is significantly influenced by diet, with age having an influence on its progression.

## 4. Impact of the Microbiome on Health 

The intestinal microbiota has been demonstrated to be involved in the regulation and maintenance of overall health. Its initial colonisation mediates immune system development and long-term colonisation determines health and survival [30]. It has influences on susceptibility to enteric, autoimmune, cardiovascular, and atopic diseases [44,50,75]. It is also involved in cognitive development and can influence subsequent cognitive disorders [9]. There is a continuous interplay between the microbiota and health as well as the impact of pathogens and diet on the microbiota. It is an interconnected, multifactorial relationship, and understanding it is crucial for optimising current practices to enhance health.

### Immune System

The intestinal microbiota has a demonstrated involvement in a myriad of functions. The interaction between the epithelial cells lining the intestine and the microbiota are essential for immune system development, maturation, regulation, and the maintenance of homeostasis [61,75,76]. Particularly, the haematopoietic and non-haematopoietic cells of the innate immune system have a unique positioning that allows them to have the ability to sense the microorganisms and their metabolic products for generation of a physiological response by the host [76]. The diversity, type, and quantity of microorganisms colonising impacts the way in which the microbiota regulates intestinal mucosal barriers, controls nutrient uptake and metabolism, assists with immune system development, and controls competitive exclusion of pathogenic microorganisms [6,30,50]. Not only do the microorganisms within the intestinal tract influence the innate immune system, they also communicate with and influence the adaptive immune system [76]. Research suggests that those animals that undergo disruptions in the microbiota or have a reduced intestinal microbial diversity are at an increased risk of cardiovascular disease, inflammatory bowel disease, necrotizing enterocolitis, eczema, obesity, malnutrition, autoimmunity, asthma, and autism [8,9,25]. Studies in germ-free animals have shown that the absence of an intestinal microbiota results in defects in lymphoid tissue development within the spleen, thymus, and lymph nodes and a reduction in lamina propria CD4+ cells, IgA-producing cells, and hypoplastic Peyer’s patches [8,20,77]. These studies have also demonstrated ileal and jejunal Peyer’s patches in pigs to be shorter at 39 and 59 days of age, predominantly T cells rather than B cells at six weeks old, and have a similar cell yield at 45 days old as a five day old normal piglet [8,20,77].

The presence of microorganisms within the GIT are clearly essential and the types of microorganisms colonising also directly influences the immune system. A good example of this is seen in the acetogenic bacteria *B. longum* subsp. *Longum* and *B. longum* subsp. *Infanis*. These species produce the short chain fatty acid, acetate, which directly influences immune system regulation by inducing regulatory T cells [78,79]. Similarities can be seen for other bacteria producing the short chain fatty acids propionate and butyrate since they have functions that inhibit the growth of pathogens. For example, acetate when administered alone inhibits the growth of *Pseudomonas aeruginosa* [80], while acetate in combination with propionate and butyrate inhibit the growth of pathogenic *E. coli* O157 [81], *Proteus mirabilis*, *Klebsiella pneumonia*, and *P. aeruginosa* [80]. This is just one example of how the microbial populations have a direct influence on immune regulation. 

In pigs, the development of the mucosal immune system occurs over a period of weeks and, from research done in germ-free pigs, it is evident that its development is largely dependent on microbial exposure [77]. This initial microbial exposure is primarily occurring at birth via urogenital and environmental exposure and at ingestion of colostrum and milk throughout lactation. Additionally, recent studies indicate that the microbiota within sow milk is dynamic and changing throughout lactation to support the piglets appropriate microbiome development, especially between the colostrum and milk phase of lactation [82]. The pig goes through a number of stressful events throughout its life and, therefore, a strong immune system is crucial for optimal growth and survival. It is understood that the stress associated with weaning, mixing of litters, and abrupt diet changes result in significant microbial shifts and dysbiosis, which reduces intestinal integrity and often leads to disease [61,83]. Dou et al. [84] demonstrated that those piglets that developed diarrhea post-weaning had a different microbiota than those who did not, with this difference being detectable as early as seven days of age (four weeks prior to diarrhea). At seven days of age, the non-diarrheic pigs displayed a higher abundance of *Prevotellaceae*, *Lachnospiraceae*, *Ruminocacaceae*, and *Lactobacillaceae* compared to diarrheic pigs. These data suggest that ensuring optimal microbial establishment in early life is essential for preventing disease during stressful periods in later life. This study did not investigate the effects of the sow or litter on this outcome. In order to understand the direct mechanisms for the differences in microbiota observed, it would be beneficial to establish if this microbial difference and susceptibility was attributed to the sow’s microbiota or the housing environment during the lactation period. Hasan et al. [85] focused on both the sow and the piglet and demonstrated that, by influencing the sow diet through yeast derivatives (YD) (brewer’s yeast hydrolysate) during pregnancy and lactation, the sow colostrum contained more fat and piglet performance was improved. Although sow fecal bacterial diversity was not different, those sows fed YD had higher levels of beneficial and fermentative bacteria and reduced numbers of opportunistic pathogenic bacteria. Furthermore, piglets from YD sows demonstrated a similar trend with increased numbers of beneficial bacteria and reduced opportunistic pathogens present in feces at one week of age. This study provides evidence that the sow’s microbiota can be manipulated in order to positively influence their offspring.

## 5. How Can We Manipulate the Microbiome to Improve Health? 

### 5.1. Prebiotics and Probiotics 

Prebiotics and probiotics are two commonly used dietary additives in both human and animal nutrition. They have been extensively studied in recent years due to their perceived health benefits. A prebiotic is a substance that is not hydrolysed or absorbed in the first part of the digestive system and reaches the colon to selectively stimulate the proliferation of resident beneficial bacterial strains [86]. Probiotics are defined as cultures of potentially beneficial bacteria of healthy gut microflora that are administered to colonise the large intestine and modify the composition of the microbiota [86,87]. To date, several reviews have investigated the effect of using both prebiotics and probiotics in treating human and animal disease [49,50,77]. 

Post-weaning diarrhea has been of particular concern to the pork industry for a long time. As such, many reviews of the literature surrounding the use of prebiotics and probiotics as a method of reducing post-weaning diarrhea have been conducted [1,58,68,83,88]. To date, the focus has been primarily around the use of prebiotics and probiotics for reducing post-weaning diarrhea, with little research surrounding their use during lactation. Hayakawa et al. [89] demonstrated that the administration of a probiotic containing *Bacillus mesentericus*, *Clostridum butyricum*, and *Enterococcus faecalis* (0.2% (*w*/*w*)) three weeks prior to farrowing and throughout lactation improved litter weight and sow return to oestrus (17% and 24% improvement, respectively). In addition to this, sow feed intake during late lactation, post weaning diarrhea incidence, and piglet growth performance were all improved. Additionally, another study found that inclusion of a prebiotic YD in a gestation and lactation diet resulted in shifts in the fecal microbiota so that the abundance of beneficial bacteria was supported and pathogenic bacteria reduced [85]. These fecal microbial differences were also associated with improved sow milk yield and piglet weight gain. To our knowledge, this is the only study that has assessed sow fecal microbial change as a result of a feed additive during lactation and its subsequent effect on offspring. From this, it is evident that prebiotics and probiotics have the potential to improve pig health. However, there is an obvious gap in the literature surrounding the use of these during the lactation period in pigs. 

### 5.2. Fecal Microbiome Transplantation

Fecal microbiome transplantation (FMT) is the transplantation of a fecal suspension from a healthy individual into the gastrointestinal tract of another individual to cure a specific disease [90]. There has been recent interest in re-establishing a “good” microbiome via competitive exclusion using FMT. One example is the case of *Clostridium difficile* infections in humans. Overgrowths of *C. difficile* have achieved epidemic proportions associated primarily, but not exclusively, with hospitalization and specific antimicrobial treatments [91]. Treatment of human *C. difficile* with antibiotics often fails as antibiotics kill vegetative bacteria but not spores. With cessation of antibiotic treatment, spores germinate and recurrent *C. difficile* disease develops. To counter this, the ability to re-establish a “good” microbiome to competitively exclude *C. difficile* using FMT has been achieved. This procedure requires that feces from a healthy donor be inoculated into the patient either orally or via an enema [92]. The use of oral FMT for treating food poisoning or severe diarrhea was first described by Ge Hong in 4th century China [90]. In recent studies, the use of FMT for treating enteric diseases induced lasting changes in the patient’s microbiome, with a >90% success rate observable within days and was without adverse side effects [90]. Brandt and Aroniadis [90] also described beneficial effects of FMT in non-enteric diseases such as Parkinson’s disease, insulin resistance, multiple sclerosis, and childhood regressive autism. 

Since antibiotic use is a growing global concern, the development of non-antibiotic techniques to treat animal disease needs to be explored. The potential of application of FMT to control preweaning and postweaning enteric diseases in pigs is intriguing. Studies by Hu et al. [93] and Xiao et al. [94] show promising results for the use of FMT within pigs. The administration of 1.5 mL of FMT daily to pigs from one to 11 days of age increased the average daily gain, reduced the incidence of diarrhea, and improved the intestinal barrier and immune system function [93]. Furthermore, positive outcomes were evident when performing FMT, with FMT from a colitis-resistant breed to an at-risk breed, which results in the improved resistance being transferred [94]. However, contrary to the results of Hu et al. [93] and Xiao et al. [94], others have demonstrated a negative effect in piglets receiving FMT four times throughout lactation directly or piglets reared on sows receiving FMT. The pigs are lighter at 70 and 155 days and have poorer absorptive capacity and intestinal health, as demonstrated through intestinal morphology and duodenal gene expression [95]. Although the results from these studies are somewhat conflicting, they provide promise for the use of FMT in pigs. It is evident that the FMT donor chosen would also have an impact on the results obtained. Therefore, it may be that the negative results observed in the study by McCormack et al. [95] were a result of the donor animals chosen. To our knowledge, these are the only studies that have investigated the use of FMT in pigs and, from this, it is evident that this area of research provides promise.

## 6. Conclusions 

It is evident that the intestinal microbiome plays an integral role in modulating health and disease. Although a large body of evidence has identified the ways in which the microbiome influences health, there is still much to learn about how we can utilise this knowledge for preventing and treating disease in humans and animals. The findings from this review demonstrate the lack of information covering the lactation period for pigs. With the critical time period for microbiome development likely occurring prior to weaning, it is crucial that further research is invested into understanding the impact routine farm practices are having on a piglet’s microbiome. It is hoped that this review will enable access to critical information for those interested in the microbiome and enable its potential for improving herd health on the farm. 
